# New data of three rare belondirid species (Nematoda, Dorylaimida, Belondiridae) from Vietnam, with the first record and description of the male of *Oxybelondira paraperplexa* Ahmad & Jairajpuri, 1979

**DOI:** 10.3897/BDJ.2.e1156

**Published:** 2014-08-11

**Authors:** Duong Thi Anh Nguyen, Tam Thi Thanh Vu, Michael Bonkowski, Reyes Peña-Santiago

**Affiliations:** † Institute of Ecology and Biological Resources, Vietnam Academy of Science and Technology, 18 Hoang Quoc Viet, Ha Noi, Vietnam; ‡ Department of Terrestrial Ecology, Institute for Zoology, University of Cologne, Zülpicher Strasse 47b, D-50674 Cologne, Germany; § Departamento de Biología Animal, Biología Vegetal y Ecología, Universidad de Jaén. Campus “Las Lagunillas”, s/n, 23071-Jaén, Spain; | Department of Terrestrial Ecology, Institute for Zoology, University of Cologne, Zülpicher Strasse 47b, 50674 Cologne, Germany

**Keywords:** Description, nematodes, new records, Oriental region, taxonomy

## Abstract

Three rare nematode species of the family Belondiridae, originally described from and only known to occur in India are recorded for the first time in Vietnam: *Axonchium thoubalicum*, *Belondira murtazai* and *Oxybelondira paraperplexa*. It is the first report of these three genera in this country. The three species are described, including new morphological data, morphometrics and light microscope pictures. The male of *Oxybelondira paraperplexa* is collected and described for the first time. It is characterized by its 1.54 mm long body, ad-cloacal pair of genital papillae situated at 9.0 µm from the cloacal aperture, only one ventromedian supplement located at 15 µm from the ad-cloacal pair within the range of spicules, spicules slightly curved ventrad and 42 µm long (7 times as long as wide and 2 times as long as cloacal body diameter), and tail 100 µm long (*c* = 15, *c*’ = 5) and similar to that of the female.

## Introduction

Dorylaims, the representatives of the nematode order Dorylaimida, with more than 2500 valid species and more than 250 valid genera (Andrássy 2009), are one of the most important taxa among Nematoda. Their diversity has been characterized with some success in several temperate (Europe, New Zealand, South Africa and USA) and a few tropical (Costa Rica and India) areas, but it remains poorly explored or nearly totally unknown in many other territories. The study of dorylaimid fauna of southeast Asia, and more particularly of Vietnam, has received little attention as only 25 species belonging to 15 genera were identified in this country until the end of the past decade in a total of 12 contributions (see Table 1 for a summary of available data). More recent studies by Nguyen (2011), however, suggest that the Vietnamese dorylaimid fauna is significantly richer.

The information regarding the occurrence of members of the family Belondiridae Thorne, 1939 in Vietnam is especially poor as it is limited to the original description of two species of the genus *Dorylaimellus* Cobb, 1913, namely *Dorylaimellus vietnamensis* Ahmad & Sturhan, 2000 and *Dorylaimellus vietnamicus* Gagarin & Nguyen, 2004. This contribution provides new data on three known belondirid genera and species, which are recorded for the first time in the country.

## Materials and methods

Nematological surveys were conducted in three locations of Northern Vietnam: Cuc Phuong National Park, Ninh Binh Province, in August 2009; Phong Nha Ke Bang National Park, Quang Binh Province, in July 2010; Huu Lien Nature Reserve, Lang Son Province, in May 2013. Soil samples from each location consisted of 200 g of soil from up to 10 cm depth. Soil samples were kept in plastic bags and brought to laboratory. Nematodes were extracted by a modified Baermann funnel technique, killed by heat, fixed in hot formaldehyde 4%, transferred to anhydrous glycerol according to Siddiqi (1964), and mounted on glass slides for further handling.

Microphotographs were taken with a Nikon Eclipse 80i light microscope provided with differential interference contrast optics (DIC) and a Nikon Digital Sight DS-U1 camera. Specimens were deposited in the collections of the Institute of Ecology and Biological Resources (IEBR), Vietnam; the Andalusian Research Group on Nematology, University of Jaén, Spain and the Institute for Zoology, Department of Terrestrial Ecology, University of Cologne, Germany.

## Taxon treatments

### Axonchium thoubalicum

Dhanachand & Jairajpuri, 1981

#### Materials

- **Type status:** Other material. **Occurrence:** recordedBy: Nguyen T. A. D; individualCount: 4; sex: 0 male, 4 females; **Location:** country: Cuc Phuong National Park, Vietnam; stateProvince: Ninh Binh; verbatimLocality: in soil around roots of *Parashorea chinensis*, karst forest; verbatimElevation: 300-400m; verbatimLatitude: 20°19’00’’ N; verbatimLongitude: 105°36’30’’ E; decimalLatitude: 20.316666; decimalLongitude: 105.6083333; **Event:** eventDate: August, 2009; **Record Level:** collectionID: Cuc Phuong 4.1 (7); Cuc Phuong 4.1 (16); institutionCode: IEBR; collectionCode: Nematode

#### Description

**Specimens examined (n=4):** Four females in good condition (Figs 1, 2).

**Measurements:** See Table 2.

**Female:** Slender nematodes of medium size. Habitus very weakly curved ventrad upon fixation. Body cylindrical, tapering towards both ends, but more so towards the anterior one. Cuticle bearing fine transverse striations, about 2.0 µm thick at neck region, 2.0 µm at mid-body, and 8–10 µm at tail. Lateral chords 7–8 µm wide or occupying one-fifth of mid-body diameter. Lip region cap-like, offset from adjacent body by a constriction, twice as wide as high and less than one-fifth (16–18%) of body diameter at neck base; lips separate, their inner portion forming liplets; papillae low, hardly protruding. Amphid fovea cup-shaped, its opening at level of the cephalic constriction and occupying 6 µm or *ca* three-fourths of lip region diameter. Odontostyle fusiform, as long as lip region diameter, with aperture occupying one-third of its total length. Guiding ring simple but distinct, located at 9 µm or 1.1 times the lip region diameter from the anterior end. Odontophore simple, rod-like. Pharynxbipartite, consisting of a slender muscular anterior section, which bears a minute (but perceptible) mucro at its beginning (observed in the four specimens examined); a deep constriction separating both sections; basal expansion nearly cylindrical, occupying 63–71% of total neck length and surrounded by a distinct spiral muscular sheath. Cardia conoid to cylindroid. Genital system mono-opisthodelphic, with the anterior branch reduced to an uterine sac *ca* twice the body diameter long whereas the posterior one is well developed: reflexed ovary does not reach the oviduct-uterus junction, oocytes first in two rows and then apparently in a single row; oviduct joining the ovary sub-terminally and consisting of a slender portion with prismatic cells and a moderately developed *pars dilatata* with distinct lumen; conspicuous sphincter between oviduct and uterus; uterus long, tripartite, consisting of a proximal wider region, narrower and longer intermediate section and a nearly sphaerical distal part; vagina 20–23 µm long, extending inwards *ca* one-half of body diameter, with *pars proximalis* surrounded by a very perceptible sphincter, *pars refringens* totally absent and *pars distalis* well developed; vulva a transverse slit. Prerectum long, 4.7–5.2 anal body widths long. Rectum shorter, 0.8–0.9 times anal body width. Tail short and rounded.

**Male:** Not found.

#### Distribution

*Axonchium thoubalicum* Dhanachand and Jairajpuri 1981 was collected in Cuc Phuong National Park, in soil around roots of *Parashorea chinensis* in karst forest.

#### Taxon discussion

This species is  known to occur onlyin India, from where it was originally described on the basis of three females and one male, and later reported by Gambhir and Dhanachand (1990), who provided measurements of two females and two males. The Vietnamese material herein examined perfectly fits the general morphology of the type material (females) (unfortunately, male was not collected in Vietnam), especially concerning the genital system. Moreover, the morphometrics of the two Indian populations and the Vietnamese one largely overlap in spite of the few number of available specimens in the three cases. The ranges of several ratios and measurements, however, are significantly widened, for instance *c* = 50–70 *vs* 53–55 in type material, *V* = 47–57 *vs* 52–54, etc. Thus, no reasonable uncertainty persists on the identity of this material.

#### Notes

This is the first record of this genus and this species in Vietnam, which might display a Oriental biogeographical range.

### Belondira murtazai

Siddiqi, 1968

Belondira rafiqi Suryawanshi 1972, by Ferris et al. (1983), syn.

#### Materials

- **Type status:** Other material. **Occurrence:** recordedBy: Nguyen T. A. D; individualCount: 4; sex: 3 males, 1 female; **Location:** country: Cuc Phuong National Park, Vietnam; stateProvince: Ninh Binh; verbatimLocality: in soil around roots of *Parashorea chinensis* in karst forest.; verbatimElevation: 300-500m; verbatimLatitude: 20°19’28’’ N; verbatimLongitude: 105°39’30’’ E; decimalLatitude: 20.3244444; decimalLongitude: 105.6583333; **Event:** eventDate: August, 2009; **Record Level:** collectionID: Cuc Phuong 1.1 (38); Cuc Phuong 4.3 (23); institutionCode: IEBR; collectionCode: Nematode

#### Description

**Specimens examined (n=4):** One female and three males in good condition (Fig. 3).

**Mesurements:** See Table 2.

**Adult:** Slender to very slender nematodes of small size. Habitus upon fixation nearly straight in female and slightly curved ventrad in males, especially in posterior body region. Body cylindrical, tapering towards both ends, but more so towards the anterior extremity. Cuticle thin, bearing fine transverse striations throughout the body. Lateral chords 4 µm wide, occupying ca one-fifth (20%) of mid-body diameter. Lip region continuous, tapering, somewhat truncate, 1.7 times as wide as high and *ca* one-fourth (25%) of body diameter at neck base; labial framework weakly sclerotized; lips amalgamated, with low papillae. Amphid fovea difficult to observe in the specimens examined. Odontostyle very short and narrow, but having perceptible lumen and aperture. Guiding ring simple. Pharynx consisting of a slender and weakly muscular anterior region which enlarges rather abruptly, pharyngeal expansion nearly cylindrical, occupying about one-half of total neck length and surrounded by a weak but well distinguishable spiral muscular sheath. Cardia rounded conoid, enveloped by the intestinal wall.

**Female:** Genital system mono-opisthodelphic. Anterior branch rudimentary, reduced to a uterine sac up to 1.5 times the corresponding body diameter long. Posterior branch well developed, but the detailed composition of its tract indistinguishable in the only one specimen examined. Tail rounded, slightly clavate, with the outer cuticle layer visibly thickened and showing radial striation.

**Male:** Genital system diorchic, with opposite testes. In addition to the ad-cloacal pair, situated at 5 µm from cloacal aperture, there are two ventromedian supplements, the posteriormost of which is located out of the range of the spicules, at 45 µm from the ad-cloacal pair. Spicules dorylaimoid, slightly curved ventral, 6.3 times as long as wide and 1.2 times as long as anal body diameter. Lateral guiding pieces difficult to observe. Tail short and rounded,  visibly concaveventrally, the outer cuticle layer less expanded than in the female. Caudal pores, if present, obscure.

#### Distribution

*Belondira murtazai* Siddiqi 1968 was collected in Cuc Phuong National Park, in soil around roots of *Parashorea chinensis* in karst forest.

#### Taxon discussion

Above description fits very well the original one of this species by Siddiqi (1968) and the revised one by Ferris et al. (1983), the latter based on the study of type material. A few minor differences, however, may be noted in the morphometrics of Indian and Vietnamese populations, but their ranges widely overlap, for instance slightly smaller general size (L = 0.77–0.94 *vs* 0.85–1.06 mm in type material as described by Siddiqi) and somewhat longer odontostyle (3–5 *vs* 3–4 µm). A major tentative difference between both populations is the length of the prevulval uterine sac (up to 1.5 *vs* 2.3–3.0 times the body diameter); nevertheless, the morphometrics given by Siddiqi certainly covers only a few out of the 12 female paratypes as Ferris et al., who examined two female paratypes loaned by Siddiqi, stated (p. 26) that the “anterior uterine branch is 1.7–2.0 body widths long”, and their Fig. 11E shows that this structure is hardly more than 1.5 times the body diameter. Ferris et al. (*op. cit.*) regarded *Belondira rafiqi* Suryawanshi, 1972, also recorded in India, as a junior synonym of *Belondira murtazai*, a decision that seems to be well supported and is herein followed.

#### Notes

This is the first record of this genus and this species in Vietnam, which might display a Oriental biogeographical range.

### Oxybelondira paraperplexa

Ahmad & Jairajpuri, 1979

#### Materials

- **Type status:** Other material. **Occurrence:** recordedBy: Nguyen T.A.D; individualCount: 21; sex: 1 male, 20 females; **Location:** country: Vietnam; stateProvince: Cuc Phuong National Park in Ninh Binh, Huu Lien Nature Reserve in Lang Son, Phong Nha – Ke Bang National Park in Quang Binh; verbatimLocality: Soil samples of karst forest; verbatimElevation: 300-500m; **Event:** eventDate: Ninh Binh: in August, 2009; Lang Son: in May, 2013; Quang Binh: in July, 2010; **Record Level:** collectionID: Cuc Phuong 5.1 (10); Cuc Phuong 1.1 (20); Cuc Phuong 3.2 (11); Cuc Phuong 3.2 (15); Huu Lien 15.1; PN-KB 27.1; institutionCode: IEBR; collectionCode: Nematode

#### Description

**Specimens examined (n=21):** Twenty females and one male in good condition (Figs 4, 5).

**Mesurements:** See Table 2.

**Adult:** Very slender nematodes of medium size. Habitus slightly curved ventrad after fixation. Body cylindrical, gradually tapering towards both extremities, but more so towards the posterior end. Cuticle thin, with fine transverse striations. Lateral chords 9–12 µm wide, occupying ca one-third of mid-body diameter. Lip region continuous, somewhat truncate, 1.7–2.0 times as wide as high and *ca* one-fifth (20%) of body diameter at neck base; labial framework well developed, having distinct labial and post-labial sclerotizations; lips amalgamated, with low papillae. Amphid fovea cup-shaped. Cheilostom a truncate cone, lacking any differentiation. Odontostyle rather strong, 1.2–1.4 times the lip region width long, with distinct lumen and aperture, which occupies *ca* one-fourth of its length. Guiding ring simple, located at 7 µm or one lip region diameter from the anterior end. Odontophore rod-like, 1.3 times the odontostyle length. Pharynx consisting of a slender part that enlarges gradually, and the basal expansion nearly cylindrical, occupying 50–56% of total neck length and surrounded by a distinct spiral muscular sheath. Cardia rounded conoid, as long as wide.

**Female:** Genital system mono-opisthodelphic. Anterior branch nearly lacking or reduced to a very short, vestigial sac.  Posterior branch well developed: ovary 72–90 µm long, reaching and occasionally surpassing the oviduct-uterus junction, with oocytes first in several rows and then apparently in one row; oviduct joining the ovary subterminally and consisting of a slender portion with prismatic cells and moderately developed *pars dilatata* with distinct lumen; a marked sphincter separates oviduct and uterus; uterus 60–70 µm long or 2.0–2.5 times the corresponding body diameter. Vagina 13–16 µm long or extending inwards *ca* one-half of body diameter: *pars proximalis* as long as wide, with somewhat sigmoid walls and enveloped by weak circular musculature; *pars refringens* lacking; *pars distalis* well developed. Vulva a pre-equatorial, transverse slit. Prerectum 6.3–6.5 anal body widths long. Rectum as long as one anal body width. Tail elongate, made of two sections of about equal length: the anterior onewider, and tapering gradually the posterior narrower and cylindrical, visibly clavate at the end; hyaline portion one-third to two-fifths of tail length.

**Male:** Genital system diorchic, with opposite testes. In addition to the ad-cloacal pair, situated at 9 µm from the cloacal aperture, one ventromedian supplement within the range of spicules, located at 15 µm from ad-cloacal pair. Spicules dorylaimoid, slightly curved ventrad and relatively slender, 7 times as long as wide and 2 times as long as anal body diameter. Lateral guiding pieces not well seen. Tail elongate, made of two sections of about equal length:anterior one wider and tapering gradually at both sides, posterior narrower and cylindrical, visibly clavate at the end; hyaline portion one-third to two-fifths of tail length.

#### Distribution

*Oxybelondira paraperplexa*  was collected in Cuc Phuong National Park and Huu Lien Nature Reserve in North Vietnam, and Phong Nha – Ke Bang National Park in central Vietnam, collected in soil samples from karst forests.

#### Taxon discussion

This is the first record of *Oxybelondira paraperplexa* after its original description from Manipur, India by Ahmad and Jairajpuri (1979), the the male is described for the first time. The Vietnamese females are identical to the type material, but new morphological data are herein provided and the ranges of the morphometrics appreciably widened.

## Supplementary Material

XML Treatment for Axonchium thoubalicum

XML Treatment for Belondira murtazai

XML Treatment for Oxybelondira paraperplexa

## Figures and Tables

**Figure 1a.**
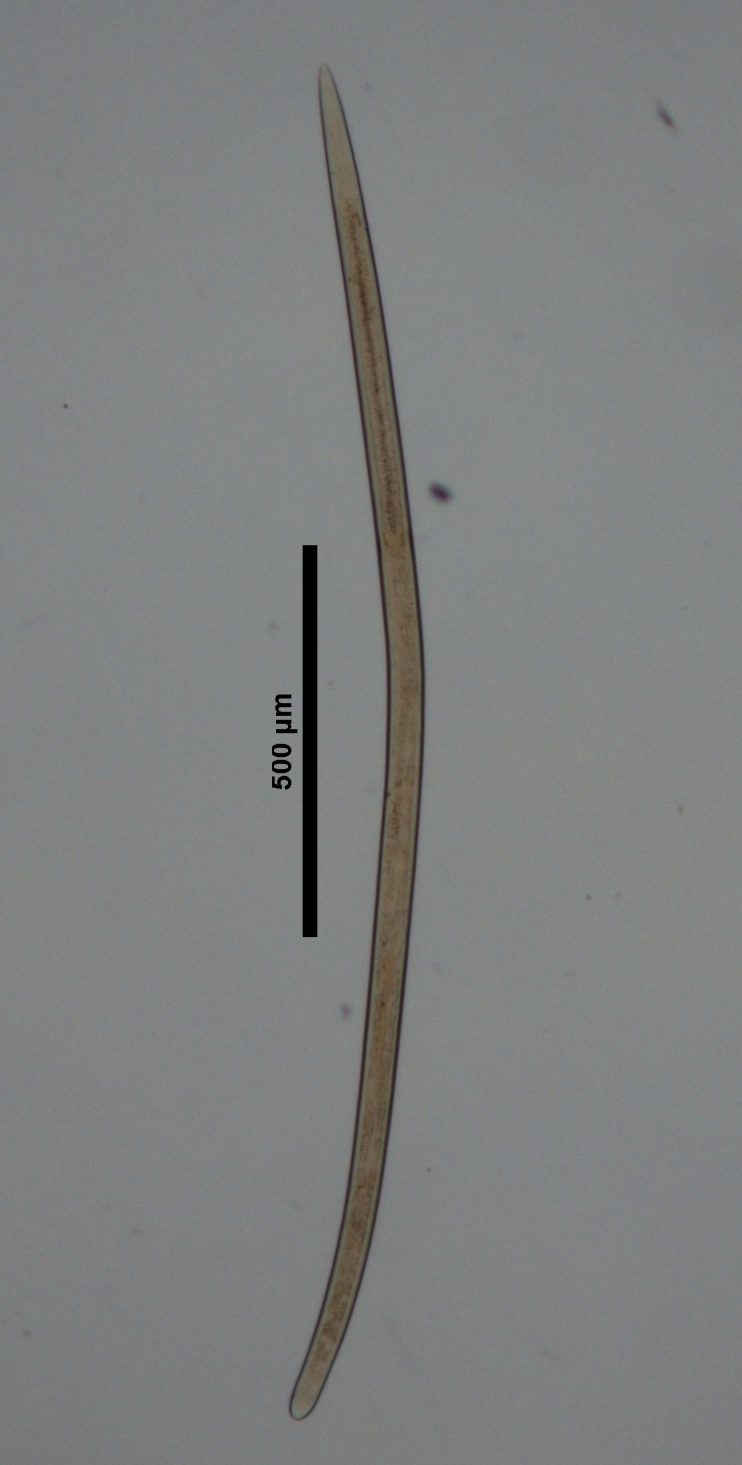
Entire

**Figure 1b.**
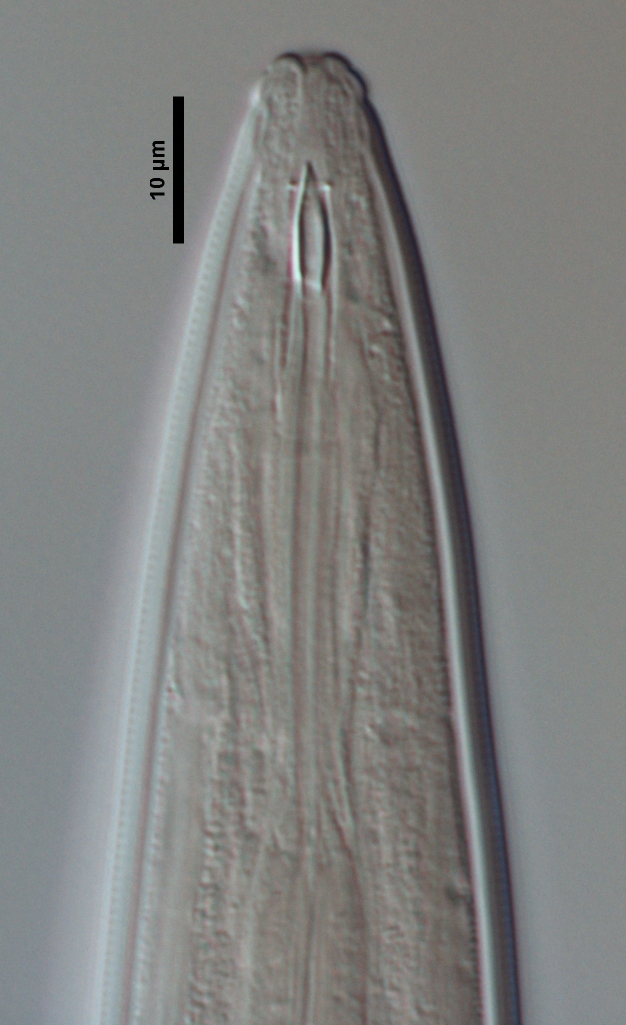
Anterior region in median view

**Figure 1c.**
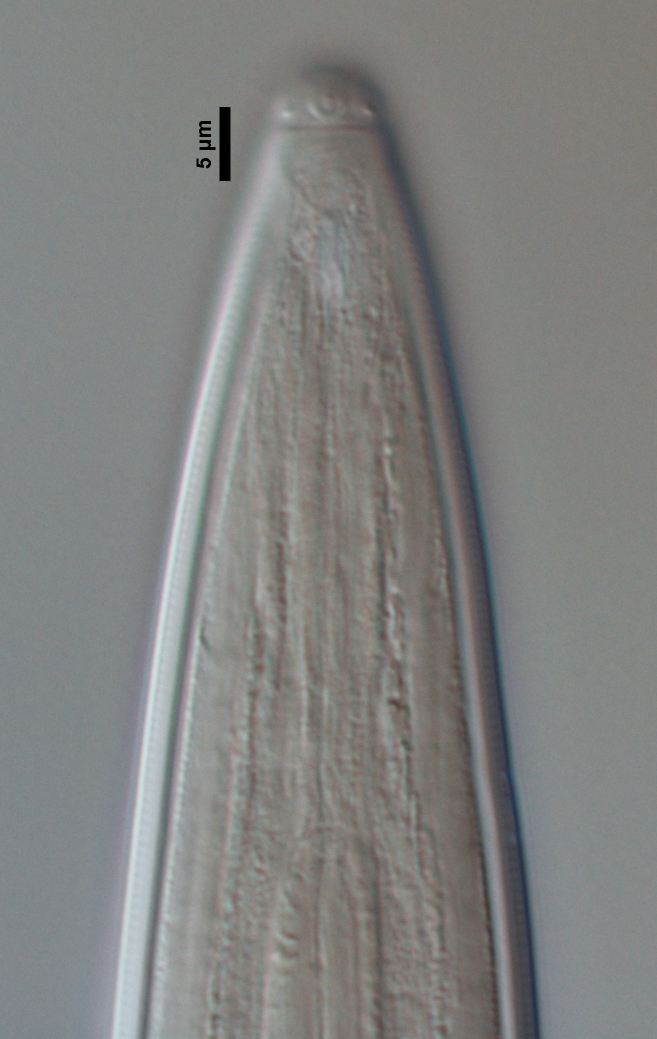
Lip region in submedian view

**Figure 1d.**
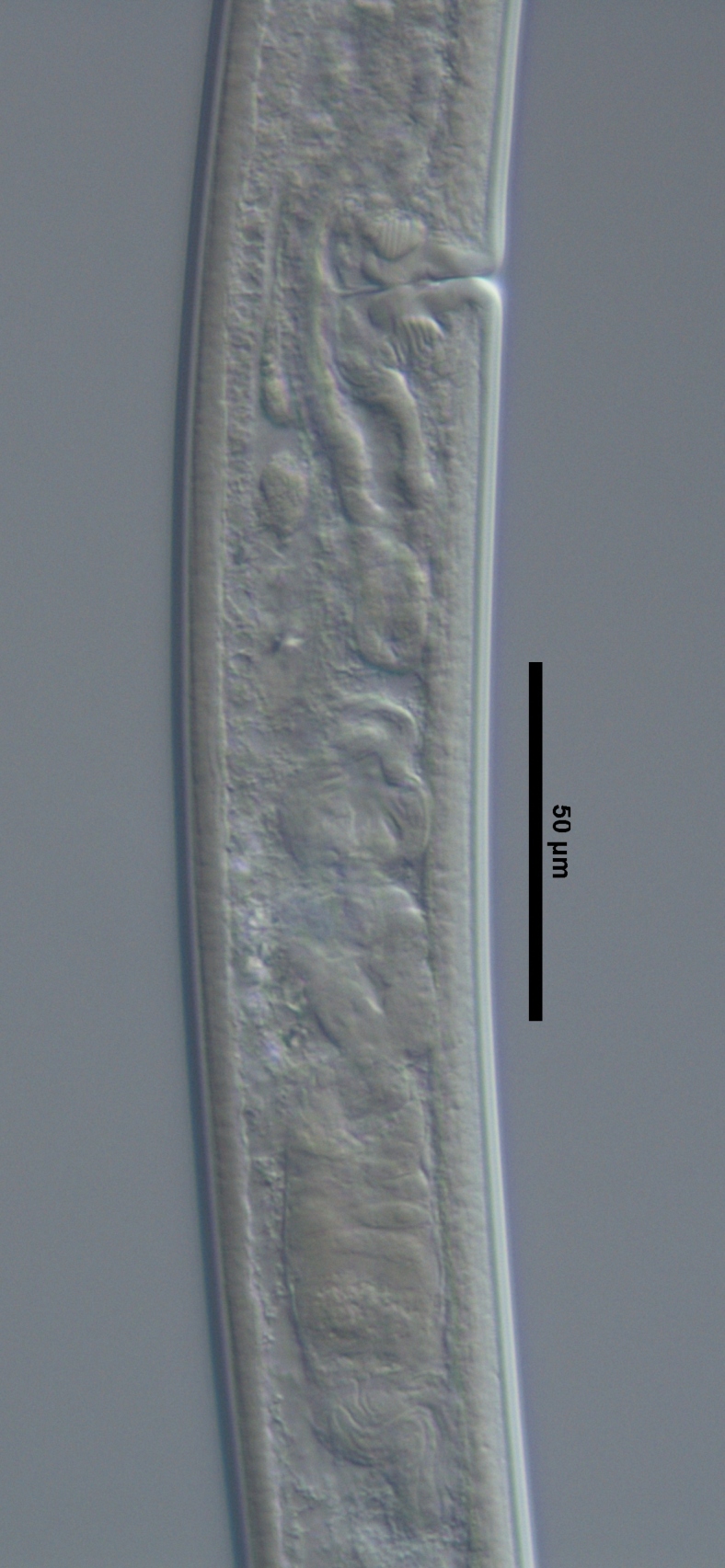
Posterior genital branch

**Figure 1e.**
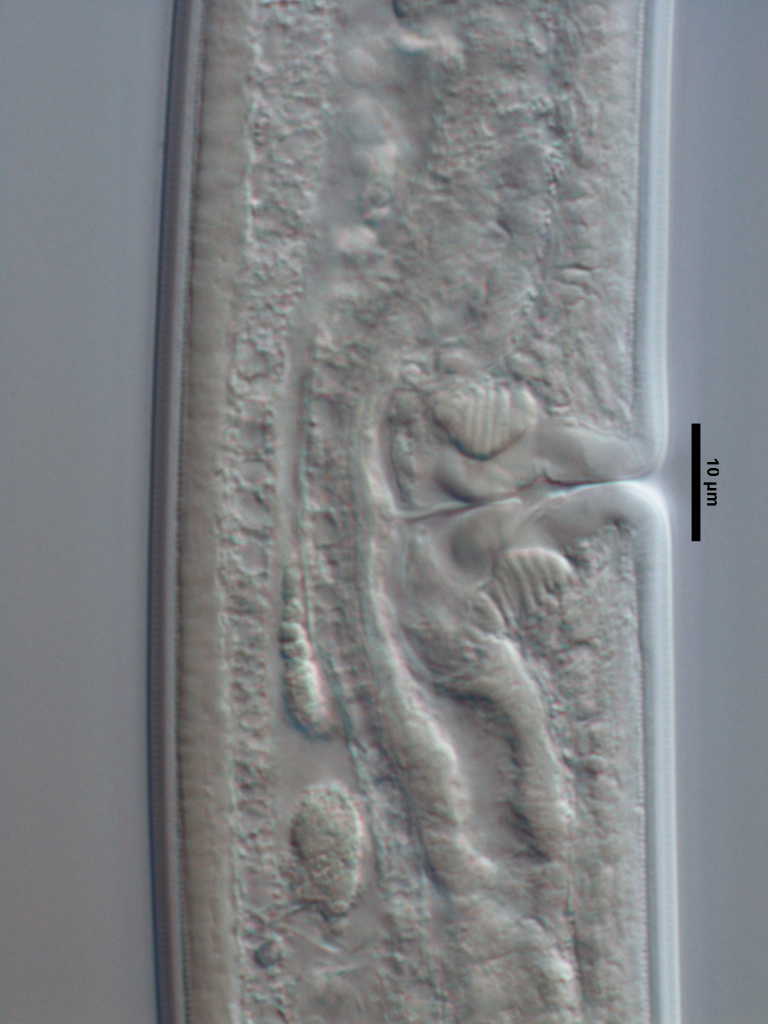
Vagina

**Figure 1f.**
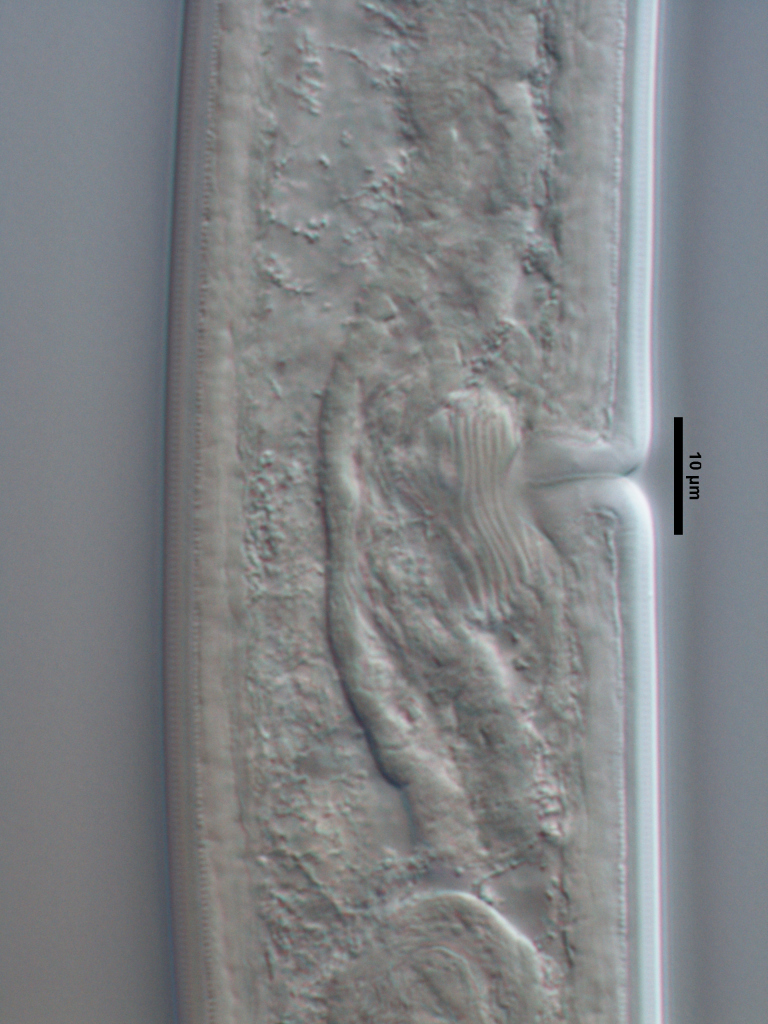
Vagina

**Figure 2a.**
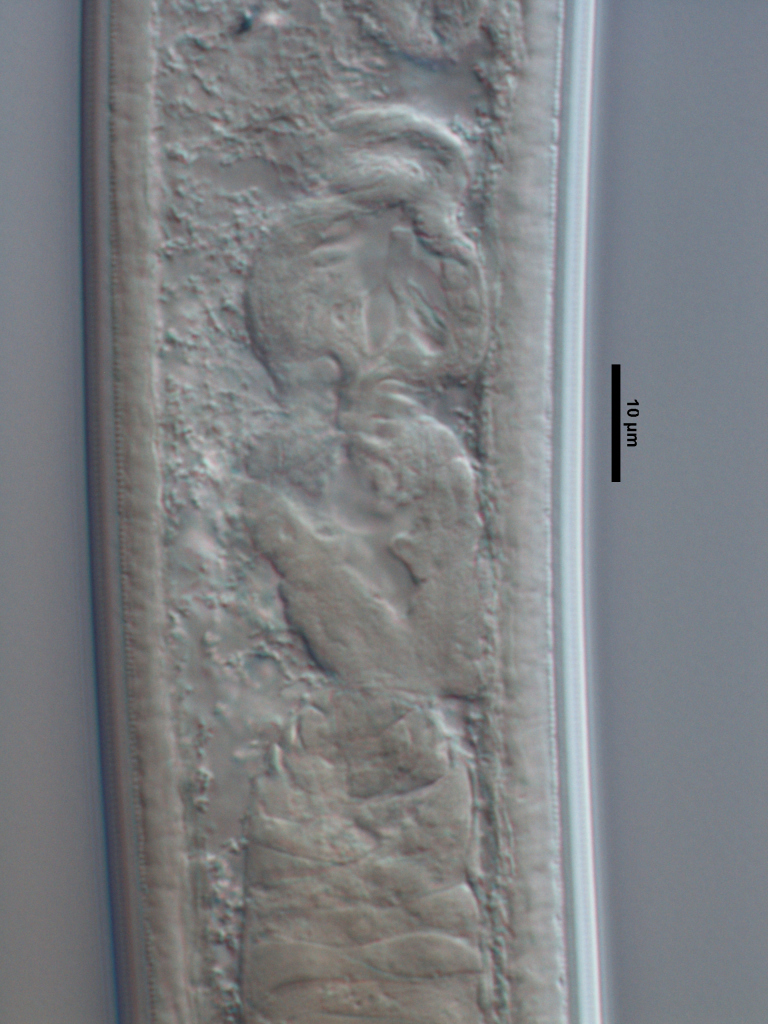
Oviduct-uterus junction

**Figure 2b.**
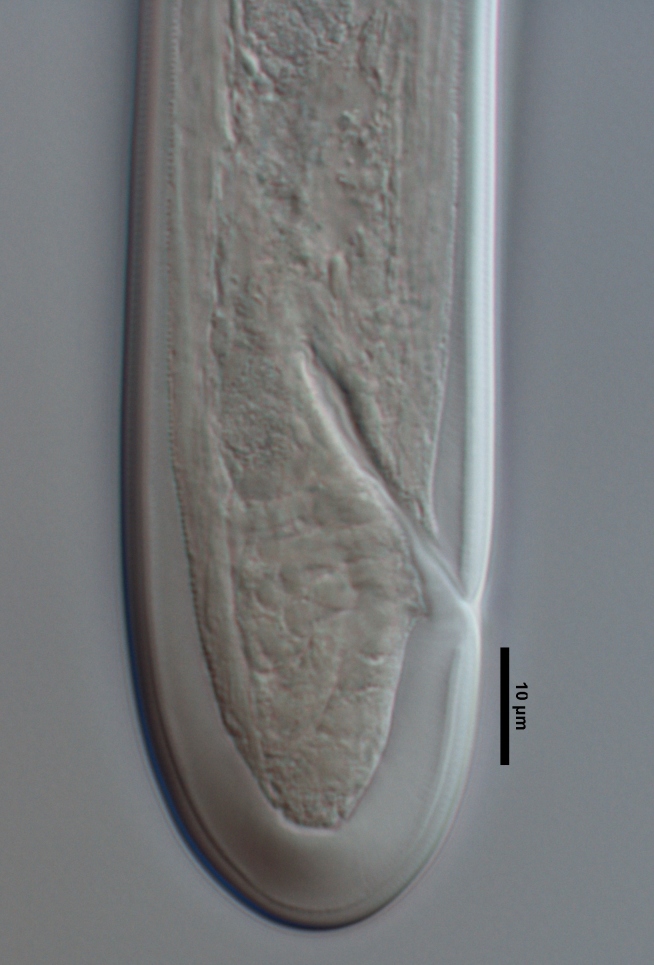
Caudal region

**Figure 3a.**
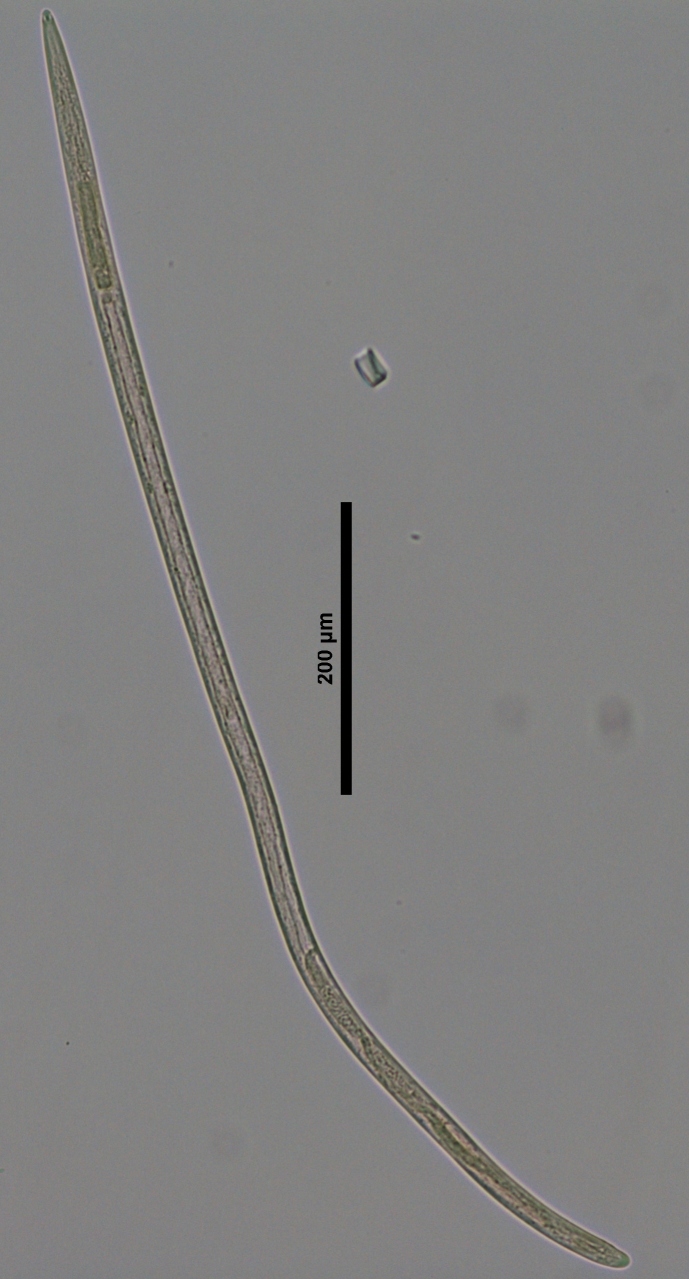
Male entire

**Figure 3b.**
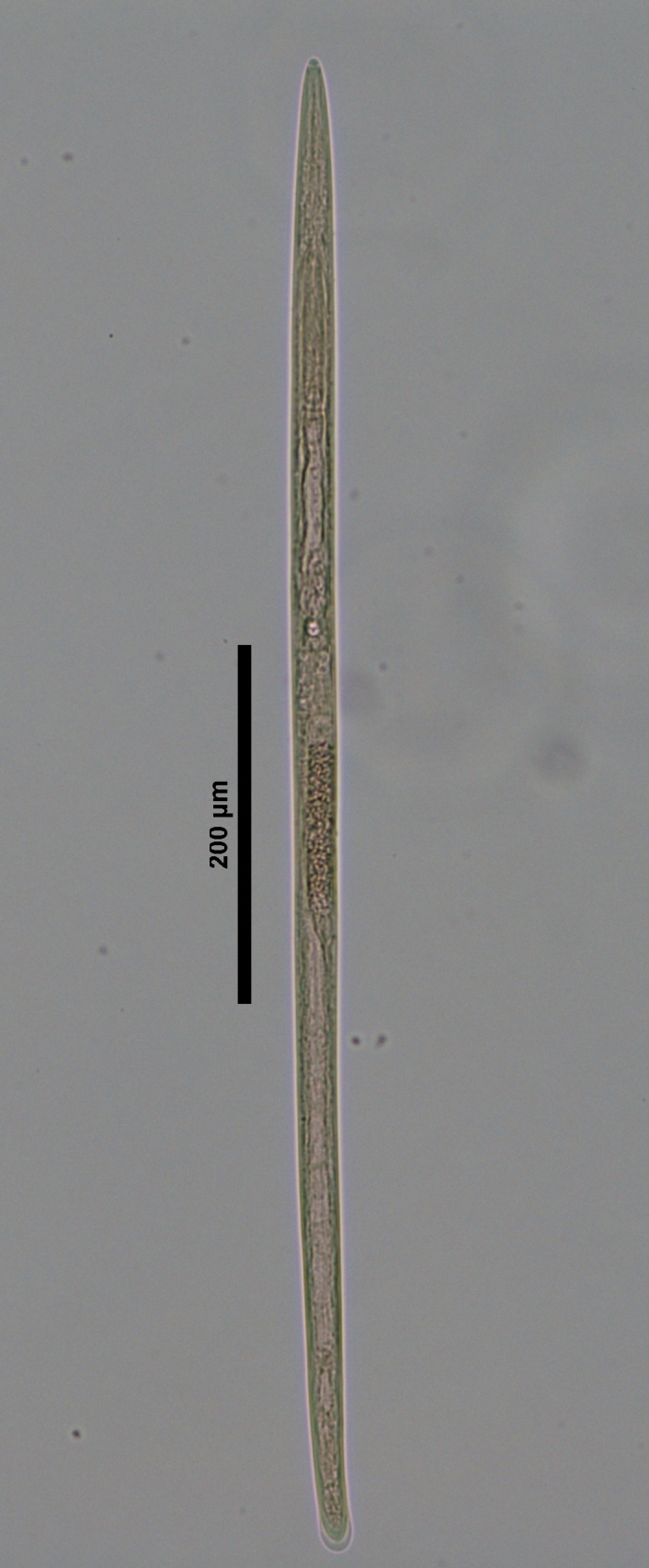
Female entire

**Figure 3c.**
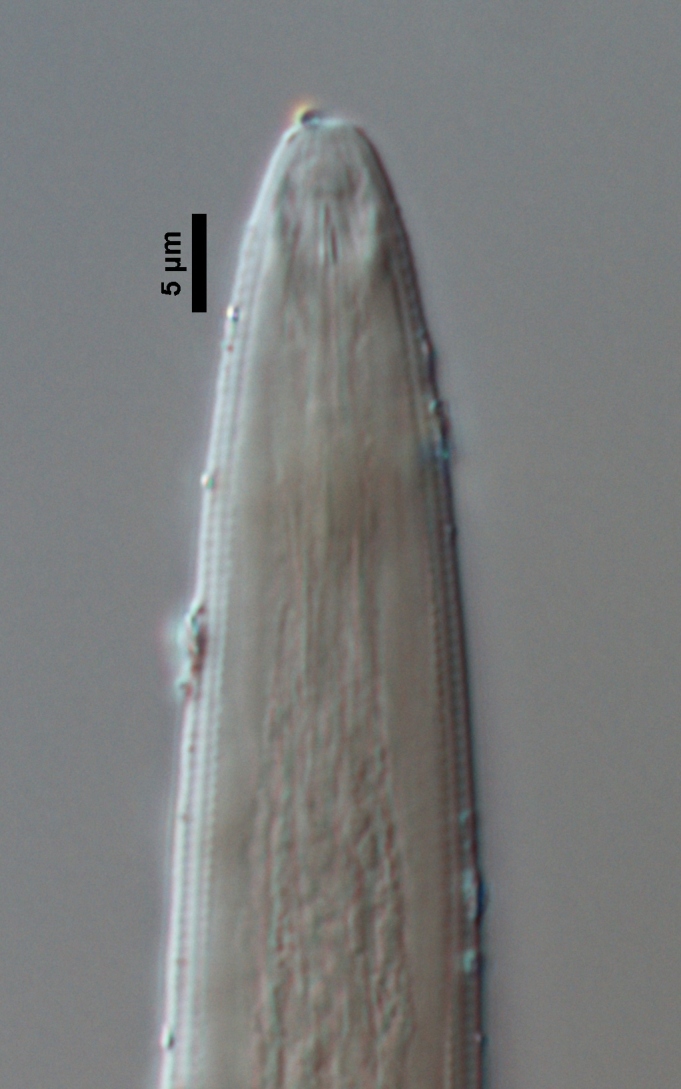
Male anterior region

**Figure 3d.**
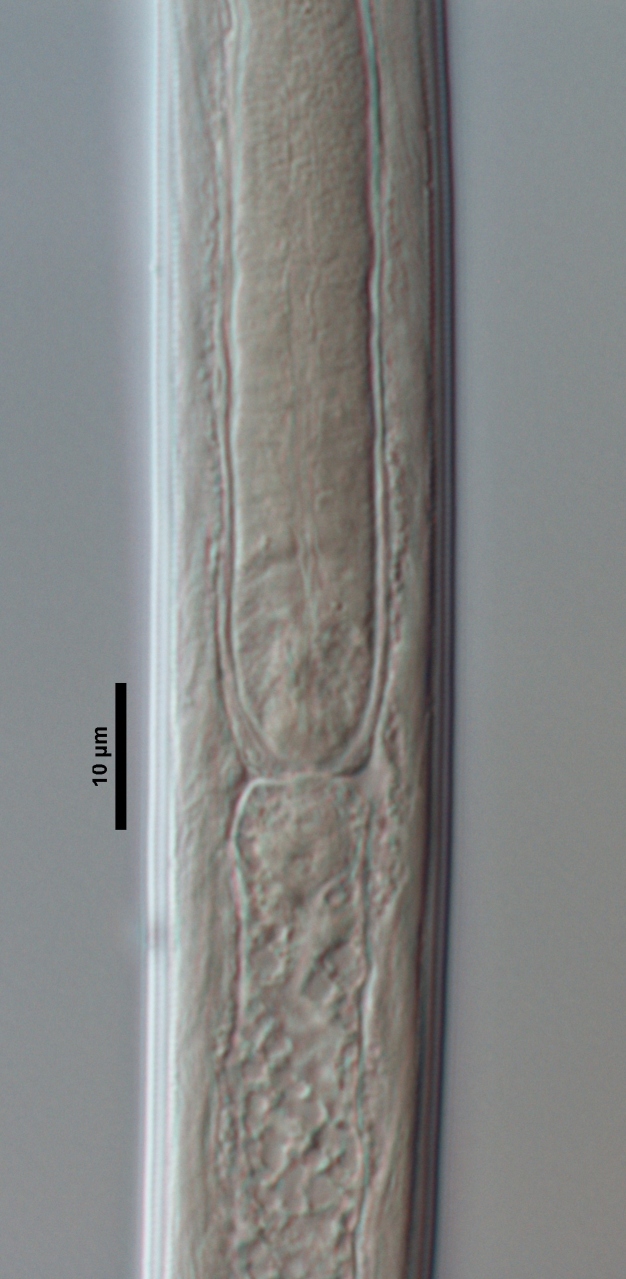
Male pharyngo-intestinal junction

**Figure 3e.**
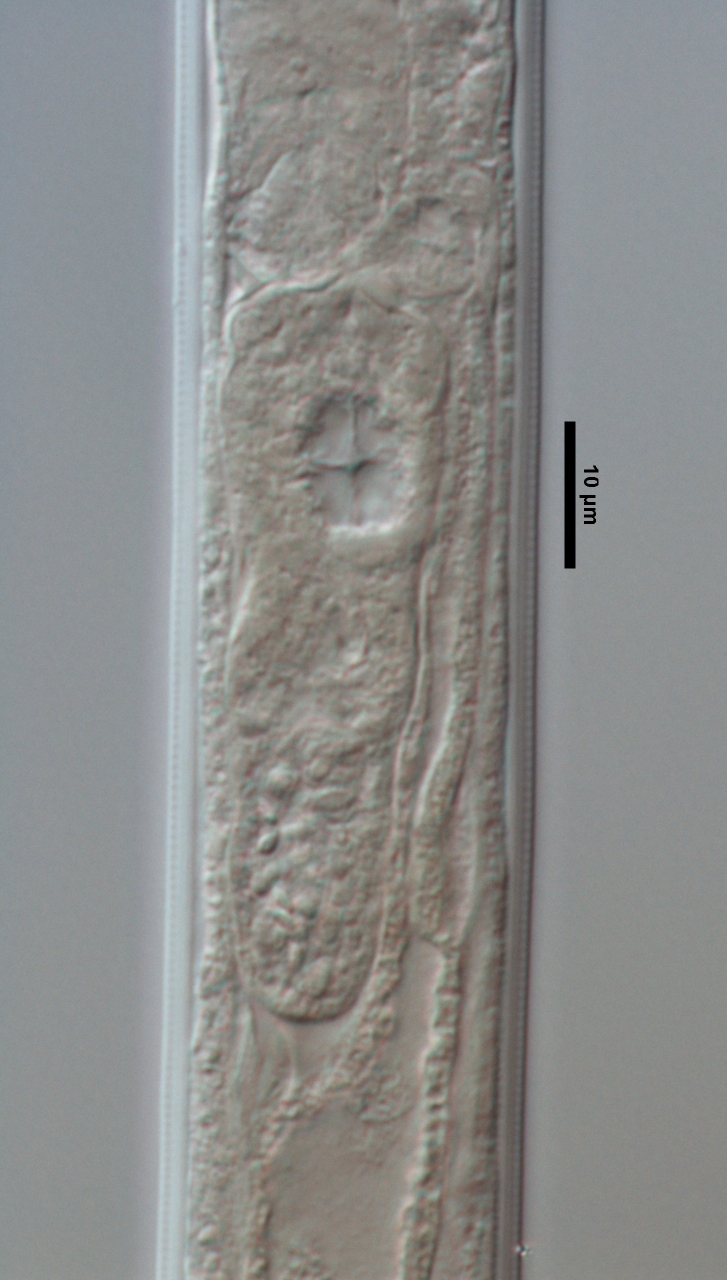
Vagina and anterior uterine sac (ventral view)

**Figure 3f.**
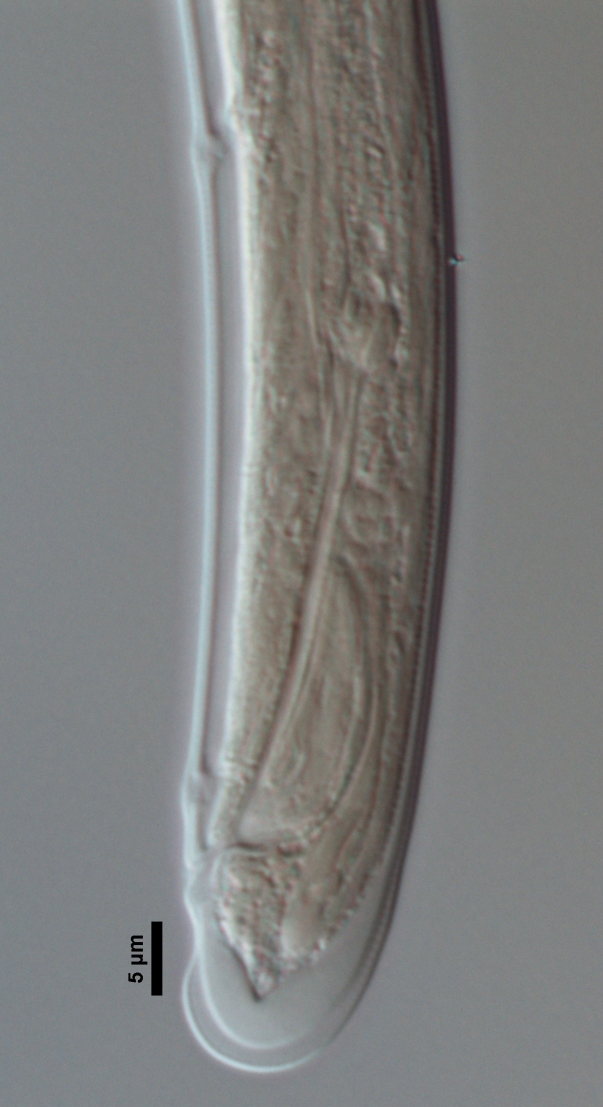
Male posterior caudal region and spicules

**Figure 4a.**
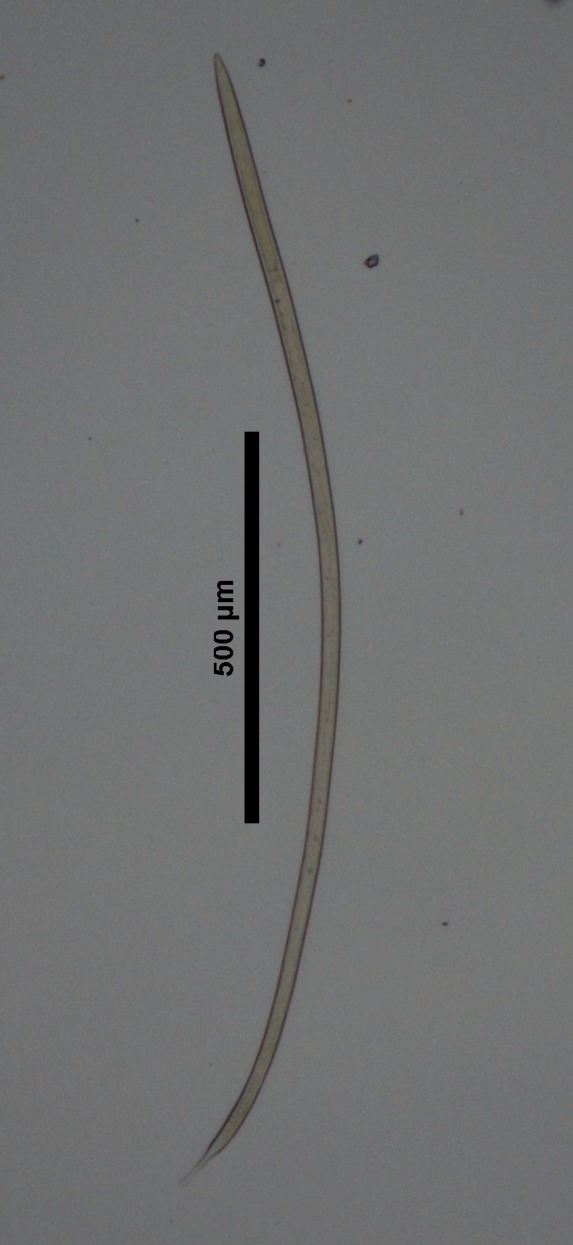
Male entire

**Figure 4b.**
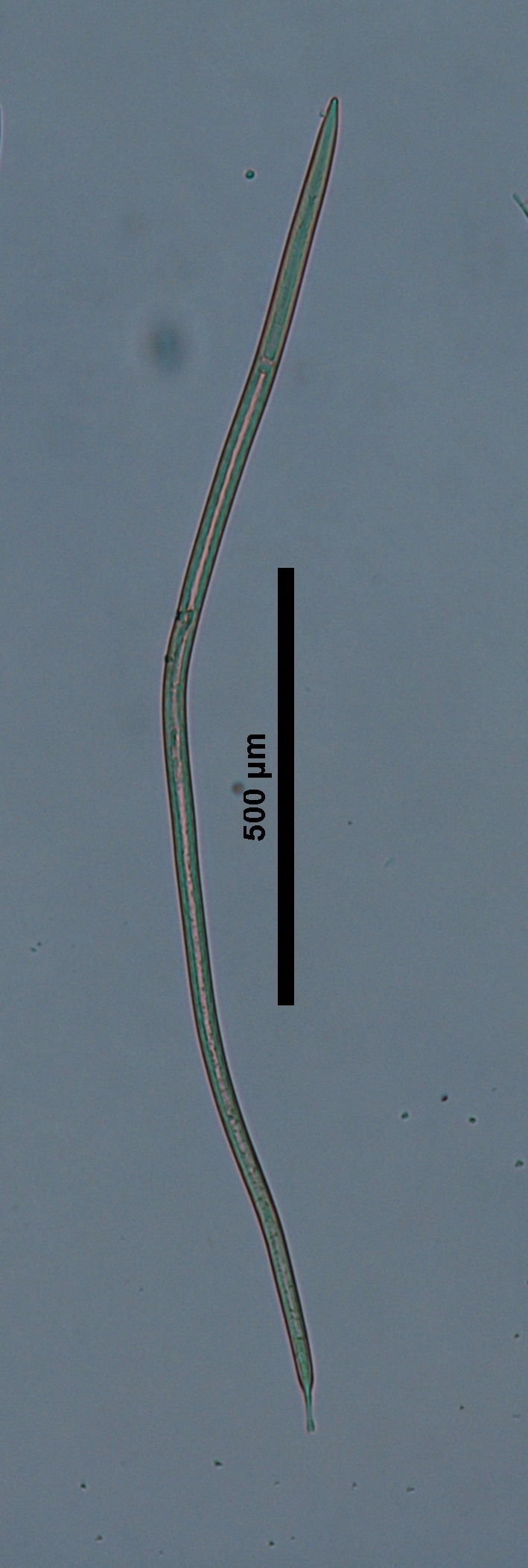
Female entire

**Figure 4c.**
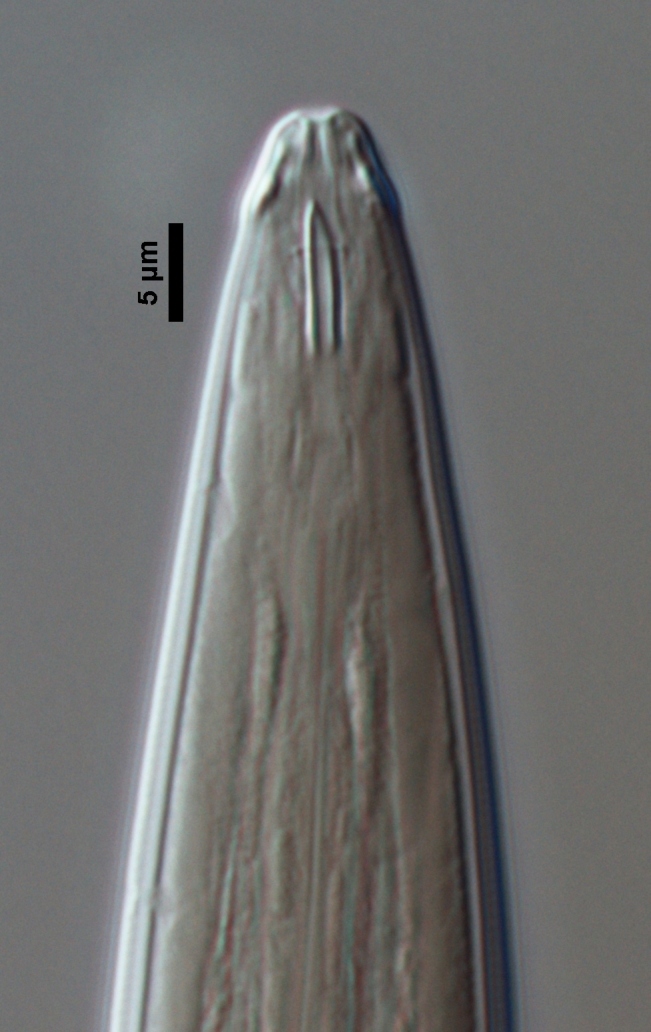
Female anterior region in median view

**Figure 4d.**
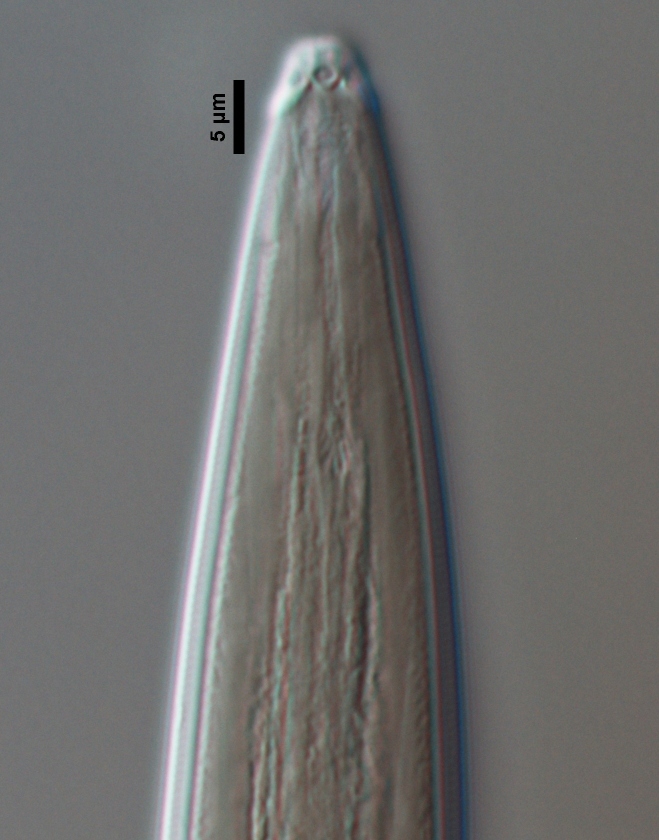
Female anterior region in surface lateral view

**Figure 4e.**
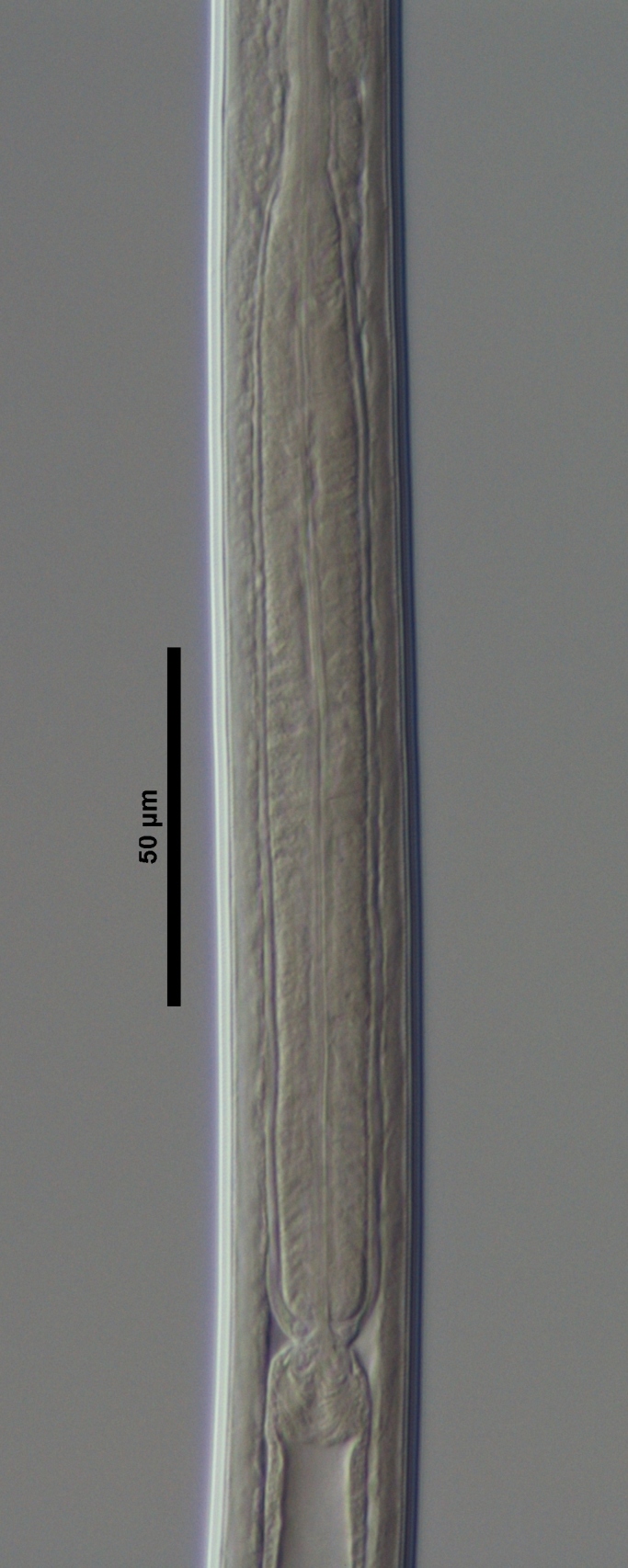
Female enlarged section of the pharynx

**Figure 4f.**
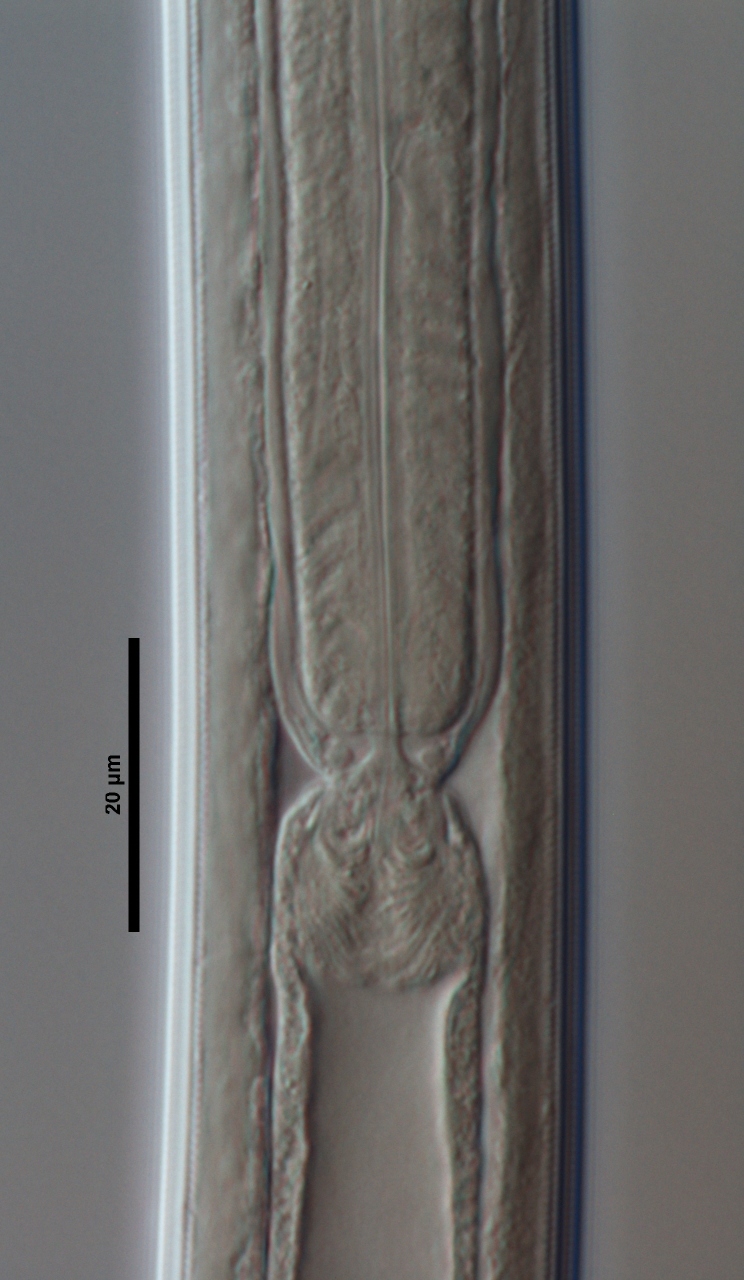
Female pharyngo-intestine junction

**Figure 5a.**
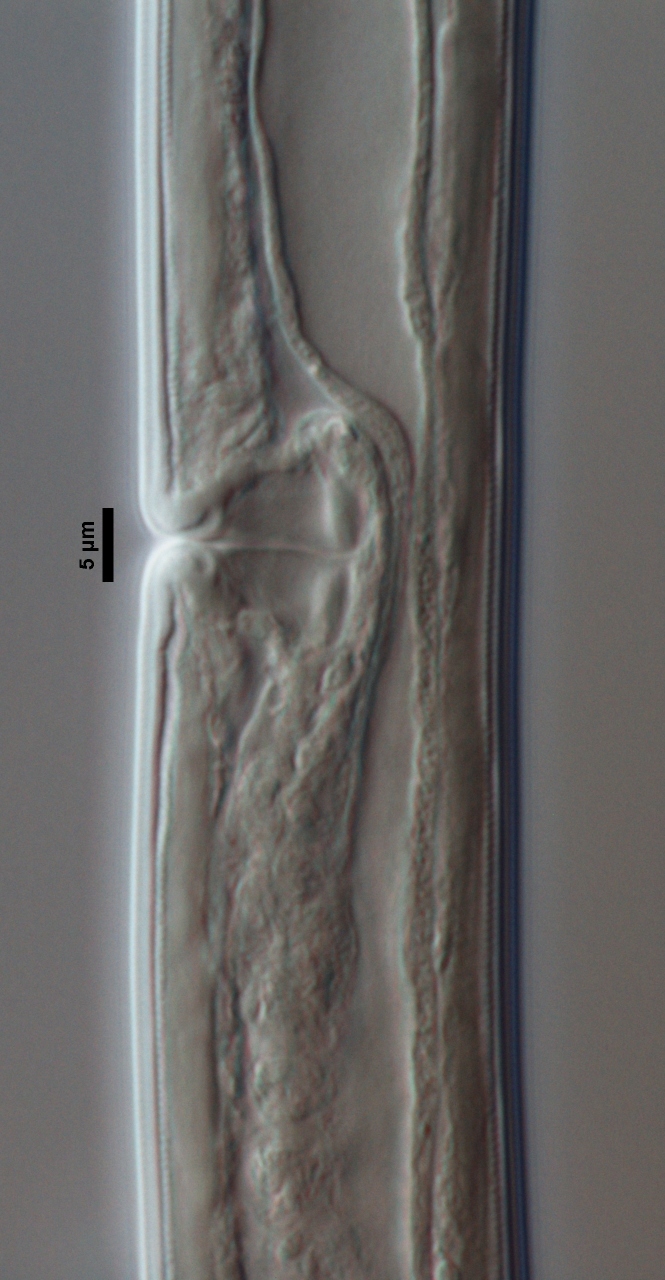
Female vagina

**Figure 5b.**
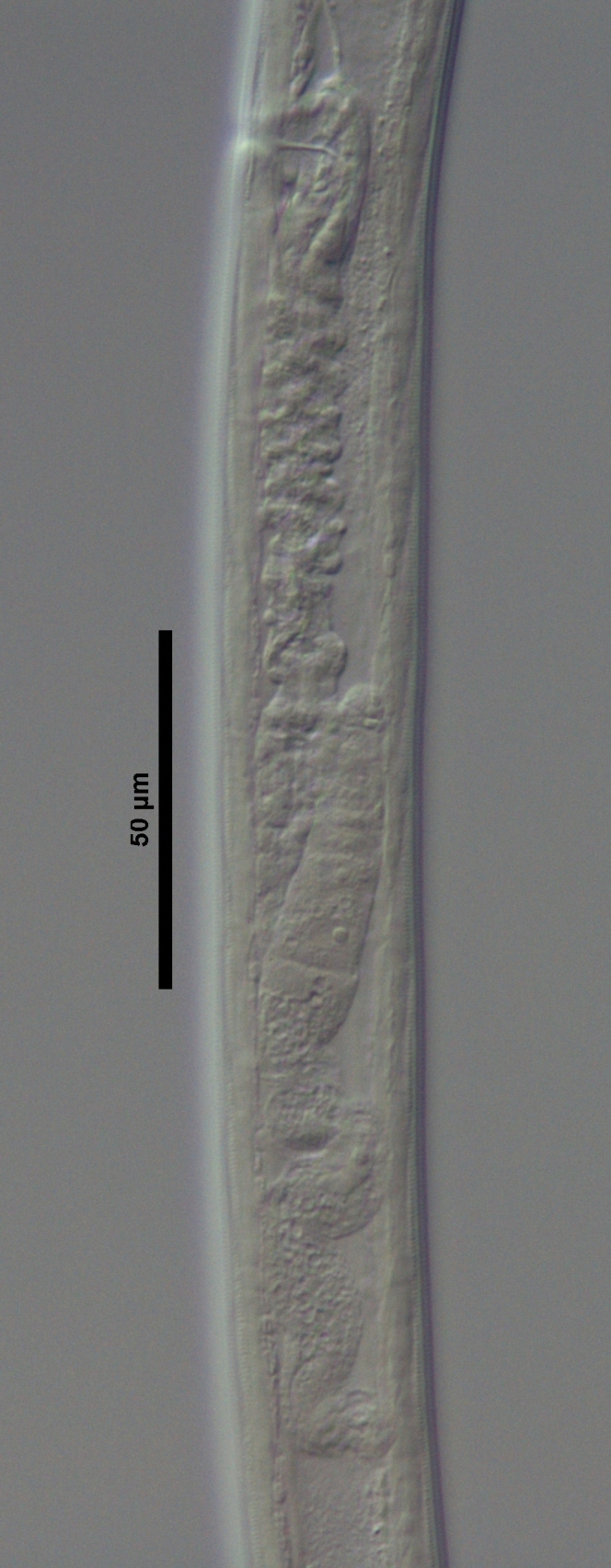
Female genital system

**Figure 5c.**
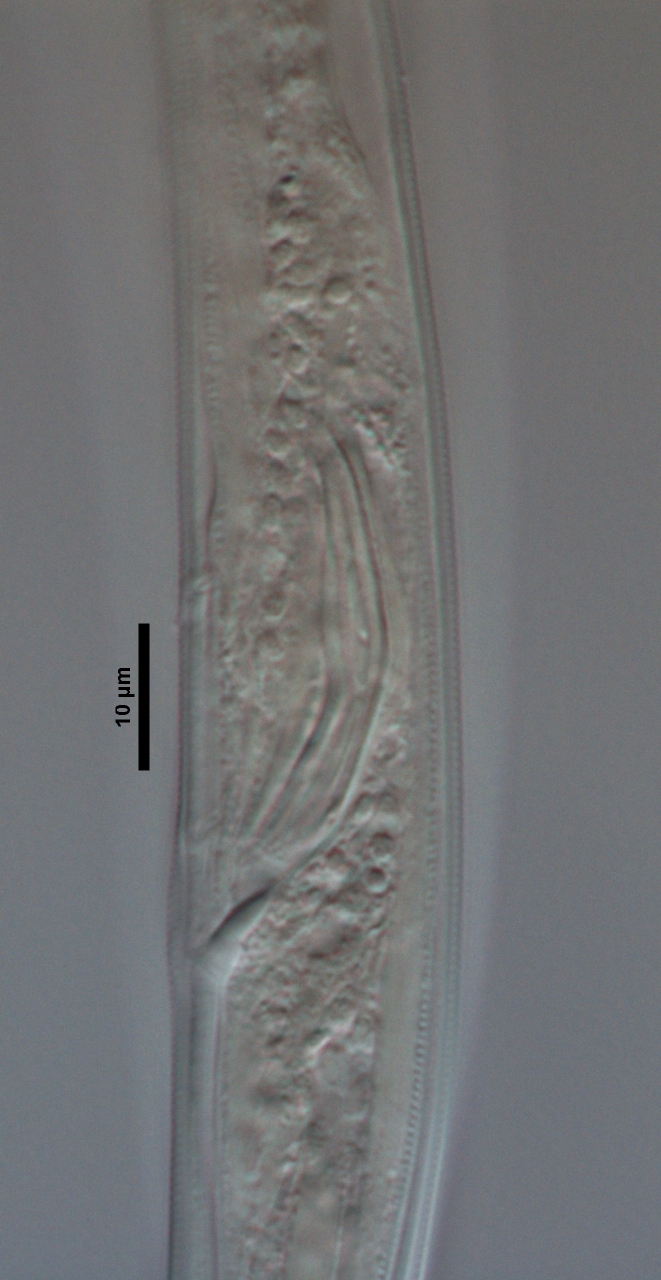
Male spicules

**Figure 5d.**
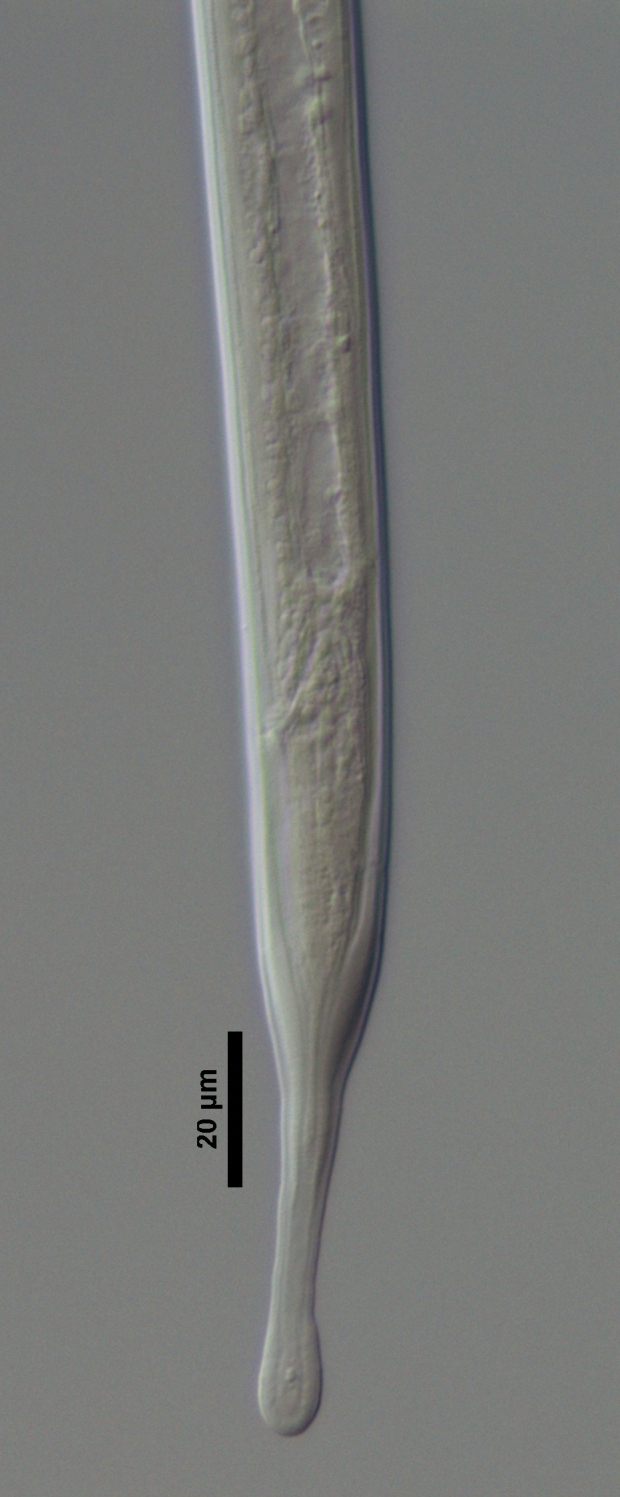
Female posterior body region

**Table 1.** Previous records of dorylaims in Vietnam.

Species	References
*Actinolaimoides angolensis* (Andrássy, 1963) Siddiqi, 1982	Andrássy (1970)
*Aporcelaimellus krygeri* Heyns, 1965	Nguyen (2007)
*Aporcelaimellus obtusicaudatus* (Bastian, 1865) Heyns, 1965	Nguyen (2007)
*Aquatides thornei* (Schneider, 1937) Heyns, 1968	Gagarin and Nguyen (2008a)
*Crassolabium aenigmaticum* Vu, Abolafia, Ciobanu & Peña-Santiago, 2010	Vu et al. (2010)
*Crassolabium vietnamense* Vu, Abolafia, Ciobanu & Peña-Santiago, 2010	Vu et al. (2010)
*Crocodorylaimus dimorphus* Andrássy, 1988	Andrássy (1988), Nguyen (2007)
*Crocodorylaimus flavomaculatus* (von Linstow, 1876) Andrássy, 1988	Nguyen (2007), Gagarin and Nguyen (2008b)
*Discolaimoides filiformis* Das, Khan & Loof, 1969	Andrássy (1970)
*Dorylaimellus vietnamensis* Ahmad & Sturhan, 2000	Ahmad and Sturhan (2000)
*Dorylaimellus vietnamicus* Gagarin & Nguyen, 2004	Gagarin and Nguyen (2004), Nguyen (2007)
*Dorylaimoides micoletzkyi* (de Man, 1921) Thorne & Swanger, 1936	Nguyen (2007)
*Dorylaimus parvus* Gagarin & Nguyen, 2003	Gagarin and Nguyen (2003), Gagarin and Nguyen (2008b)
*Dorylaimus stagnalis* Dujardin, 1845	Nguyen (2007)
*Drepanodorylaimus brevicaudatus* Andrássy, 1970	Andrássy (1970)
*Labronema neopacificum* Rahman, Jairajpuri, Ahmad & Ahmad, 1986	Álvarez-Ortega et al. (2010)
*Laimydorus oxurus* Gagarin & Nguyen, 2005	Gagarin and Nguyen (2005), Nguyen (2007)
*Laimydorus pseudostagnalis* (Micoletzky, 1927) Siddiqi, 1969	Nguyen (2007)
*Mesodorylaimus derni* Loof, 1969	Nguyen (2007)
*Mesodorylaimus lopadusae* Vinciguerra & La Fauci, 1978	Nguyen (2007), Gagarin and Nguyen (2008b)
*Mesodorylaimus lutosus* Gagarin & Nguyen, 2005	Gagarin and Nguyen (2005), Nguyen (2007)
*Mesodorylaimus mesonyctius* (Kreis, 1930) Andrássy, 1959	Nguyen (2007)
*Mesodorylaimus orientalis* Andrássy, 1970	Andrássy (1970)
*Opisthodorylaimus cavalcantii* (Lordello, 1955) Carbonell & Coomans, 1986	Andrássy (2007)
*Prodorylaimus longicaudatoides* Altherr, 1968	Nguyen (2007)

**Table 2.** Morphometrics of *Axonchium thoubalicum* Dhanachand & Jairajpuri, 1981, *Belondira murtazai* Siddiqi, 1968 and *Oxybelondira paraperplexa* Ahmad & Jairajpuri, 1979 from Vietnam. All measurements in µm except L in mm.

Species	*Axonchium thoubalicum*		*Oxybelondira paraperplexa*			
Natural Reserve	Cuc Phuong		Cuc Phuong		Phong Nha	Huu Lien
Province	Ninh Binh		Ninh Binh		Quang Binh	Lang Son
n	4♀♀	3♂♂	♂	12♀♀	2♀♀	6♀♀
Character						
L	1.63 ± 0.12 (1.50–1.75)	0.83 ± 0.09 (0.77–0.94)	1.54	1.55 ± 0.07 (1.46–1.68)	1.51, 1.77	1.44 ± 0.12 (1.43–1.45)
a	35.3 ± 1.7 (33–37)	41.1 ± 5.2 (38–47)	59.4	55.6 ± 3.9 (50–62)	44, 52	46 ± 1.7 (45–48)
b	2.5 ± 0.3 (2.2–2.8)	4.2 ± 0.5 (3.9–4.8)	5.1	5.1 ± 0.1 (4.9–5.4)	4.7, 5.1	5.2 ± 0.2 (4.9–5.3)
c	59.9 ± 10.5 (50–70)	50.4 ± 3.0 (47–53)	15	17.3 ± 4.1 (14–21)	14, 17	15.3 ± 2.0 (14–18)
V/T	53 ± 4.0 (47–57)	42 ± 9.8 (31–49)	?	39 ± 2.0 (36–41)	37, 44	39 ± 3 (36–41)
c’	0.8 ± 0.1 (0.7–1.0)	1.1 ± 0.2 (0.9–1.3)	5	4.5 ± 1.1 (2.0–5.5)	5.1, 5.4	5.1 ± 0.9 (4.1–5.9)
Lip region diameter	8	5	8	8	8	7
Odontostyle length	9	4.0 ± 1.0 (3–5)	8	8	8	8
Odontophore length	10.5 ± 0.6 (10–11)	?	9	9	8	9
Neck length	645 ± 30 (630–690)	198 ± 2.5 (19–200)	302	306 ± 11 (295–335)	324, 348	280 ± 95 (274–291)
Pharyngeal expansion length	425 ± 44 (400–490)	86.7 ± 5.8 (80–90)	150	157 ± 10 (145–175)	170, 175	155 ± 5 (150–160)
Body diam. at neck base	47.0 ± 2.4 (45–50)	21.3 ± 0.6 (21–22)	28	28.5 ± 2.0 (25–32)	28	27.7 ± 2.5 (25–30)
Body diam. at mid-body	46.0 ± 1.4 (45–48)	20.3 ± 0.6 (20–21)	26	28.0 ± 2.4 (24–32)	32	31.0 ± 1 (30–32)
Body diam. at cloaca	33.0 ± 2.4 (30–35)	15.7 ± 0.6 (15–16)	20	19.8 ± 0.6 (18–20)	20	18.7 ± 1.1 (18–20)
Prerectum length	160 ± 8.7 (150–165)	?	?	120 ± 20 (80–130)	80, 100	65 ± 13.2 (55–80)
Rectum length	27.8 ± 1.5 (27–30)	?	?	21.7 ± 2.8 (18–25)	20, 22	40 ± 0 (40–40)
Tail length	27.5 ± 2.9 (25–30)	16.7 ± 2.9 (15–20)	100	96.8 ± 6.3 (90–110)	101, 108	95 ± 12 (82–106)
Spicules length	-	20	42	-	-	-
Ventro median supplements	-	2	1		-	-
